# The N-terminus of mature human frataxin is intrinsically unfolded

**DOI:** 10.1111/j.1742-4658.2009.07381.x

**Published:** 2009-11

**Authors:** Filippo Prischi, Clelia Giannini, Salvatore Adinolfi, Annalisa Pastore

**Affiliations:** 1 Dipartimento di Biologia Molecolare, University of SienaSiena, Italy; 2 Dipartimento di Chimica Organica ed Industriale, University of MilanoItaly; 3 National Institute for Medical ResearchMRC, The Ridgeway, London, UK

**Keywords:** dynamics, Friedreich's ataxia, IUPs, NMR, structure

## Abstract

Frataxin is a highly conserved nuclear-encoded mitochondrial protein whose deficiency is the primary cause of Friedreich’s ataxia, an autosomal recessive neurodegenerative disease. The frataxin structure comprises a well-characterized globular domain that is present in all species and is preceded in eukaryotes by a non-conserved N-terminal tail that contains the mitochondrial import signal. Little is known about the structure and dynamic properties of the N-terminal tail. Here, we show that this region is flexible and intrinsically unfolded in human frataxin. It does not alter the iron-binding or self-aggregation properties of the globular domain. It is therefore very unlikely that this region could be important for the conserved functions of the protein.

## Introduction

Friedreich’s ataxia, the most common hereditary ataxia, is a progressive neurodegenerative disease caused by a large expansion of the trinucleotide repeat GAA within the first intron of the *X25* gene on human chromosome 9q13 [1]. The expanded repeat causes severe reduction in the amount of the corresponding mRNA and consequently in the amount of frataxin [2,3], a highly conserved nuclear-encoded mitochondrial protein that has been implicated in iron metabolism [4–7]. However, the function of frataxin is still not clearly established. Frataxin has been suggested to act as an iron chaperone [8] or as a scavenger that is able to sequester mitochondrial iron through formation of high-molecular-weight aggregates and to maintain it in a bioavailable form [9]. More recently, we have shown that CyaY, the frataxin bacterial orthologue, is an inhibitor of the iron–sulfur cluster enzymatic machinery, and proposed that this function is shared by all members of the family [10]. To validate these hypotheses, it is important to identify the functionally essential regions of the protein and show that they are conserved throughout the frataxin family. Regions that are not conserved may explain species-specific specialized functions.

Two distinct regions can be identified in the frataxin sequence: an N-terminus that is absent in bacteria, and a C-terminal domain that is present in all species from bacteria to human, which folds into a globular domain. The structure of the evolutionarily conserved domain of various species has been described [11–15]. However, little remains known about the eukaryotic N-terminus, which has been suggested to perform a structural gating role that would inhibit the iron-promoted binding of frataxin to IscU, a scaffold protein involved in the iron–sulfur cluster assembly [16].

In this paper, we report on a study of the structural and dynamic properties of the N-terminus of human frataxin (hfra). We have studied both the mature form, which spans residues 81-210 of the precursor protein [17,18], and a longer construct comprising residues 61-210. Longer forms of the protein are interesting as mitochondrial maturation occurs via a two-step mechanism that first generates a 19 kDa intermediate comprising residues 42-210, which further matures to the final species [17,18]. Other truncated forms, such as 56-210 and 78-210, are also present *in vivo* when the normal maturation process of frataxin is impaired [18].

Based on several independent parameters, we show that the N-terminal extension behaves in solution as a flexible and intrinsically unfolded tail that does not influence the properties of the nearby domain.

## Results

### The N-terminus of eukaryotic frataxins is non-conserved

**Fig. 1:**
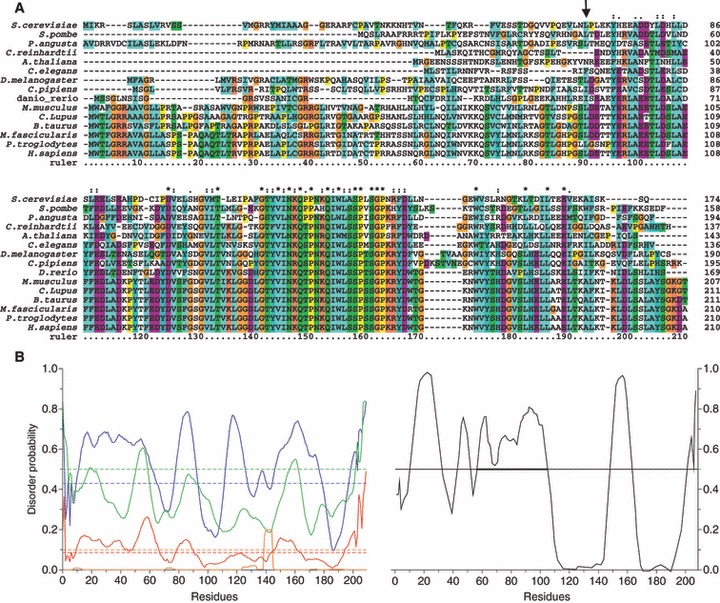
Sequence analysis. (A) Alignment of eukaryotic frataxins, colour-coded to highlight sequence conservation. An arrow indicates the start of the evolutionarily conserved globular domain. Asterisks, colons and dots indicate identical, conserved, and semi-conserved residues, respectively. The alignment was prepared using clustal x software [20]. (B) Predictions of intrinsically unfolded regions along the sequence of the hfra precursor (residues 1-210). Left panel: DISEMBL prediction [21]. The green curve is the prediction for missing coordinates, the red curve indicates the hot loop network, and the blue curve indicates coil according to the DISEMBL definition [21]. Horizontal lines correspond to the random expectation level for each predictor. For coils and hot loops, the prior probabilities were used, and a neural network score of 0.5 was used for REMARK465. Right panel: PONDR prediction [22]. The predicted disordered regions are those with higher positive values.

We first analysed the degree of conservation of frataxins among eukaryotes. We BLAST-retrieved and aligned sequences from yeast or unicellular algae to those of *Homo sapiens* (Fig. 1A) [19,20]. The alignment is straightforward as the sequences share 30–50% identity and up to 80% similarity, with almost no insertions/deletions in the evolutionarily conserved domain. Sequence conservation decreases at the N-terminus, which contains the mitochondrial import signal. The secondary structure predictions correctly identify the structural elements in the domain, and suggest the presence of potentially structured regions in the N-terminus but with low confidence (i.e. below 50%).

Interestingly, large portions of the N-terminal extension were predicted to be intrinsically unfolded by the widely used DISEMBL and PONDR web servers (http://dis.embl.de/ and http://www.pondr.com/) [21,22] (Fig. 1B). The programs also predict two regions in the evolutionary conserved domain to be intrinsically unfolded. The first is around residues 145–165 and contains the well-structured and rigid β-strands β3–β5. This is presumably because this region contains two potentially low-complexity sequences (two hydrophobic stretches interleaved by an SSPSSG sequence rich in small amino acids). The second comprises the C-terminus, which does not adopt a regular conformation, but is rather rigid as it is sandwiched between the two helices.

### The N-terminal tail of hfra does not alter the structure of the globular domain

We studied experimentally the behaviour of three constructs. One [hfra(81-210)] corresponds to the mature form [17,18]. The second spans residues 61-210 [hfra(61-210)]. As described previously [11], unless otherwise protected, this construct degrades spontaneously to produce a fragment comprising residues 75-210 [hfra(75-210)]. However, we found that use of the Complete anti-protease cocktail (Roche) prevents proteolytic cleavage. This enabled us to develop a method for comparing the behaviour of the three constructs that provides information about the structure of both the mature and the precursor forms.

**Fig. 2:**
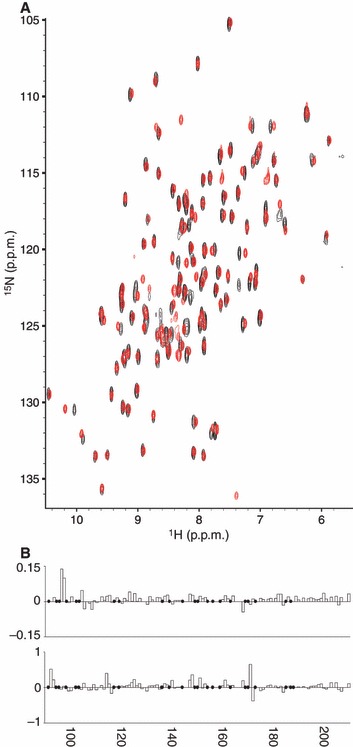
Comparison of the NMR chemical shifts of hfra(81-210) and the evolutionarily conserved globular domain hfra(91-210). (A) Superposition of the ^15^N HSQC spectra of hfra(81-210) (in black) and hfra(91-210) (in red). (B) Plots of the chemical shift differences between the two constructs in the nitrogen dimension (lower panel) and proton dimension (upper panel) as a function of the sequence.

The HSQC spectrum of hfra(81-210) is largely superimposable with that of a shorter construct, hfra(91-210), described in a previous study [11], that spans the sequence of the evolutionary conserved domain (Fig. 2A). Only minor local differences (i.e. less than 0.14 and 0.6 ppm in the proton and nitrogen dimensions, respectively) were observed (Fig. 2B). The differences cluster in the regions spatially close to Asp91, indicating the absence of specific interactions between the N-terminal extension and the domain. Therefore, the N-terminal tail has no influence on the structure of the conserved domain. The spectrum of hfra(81-210) contains seven clearly identifiable additional peaks that are absent in hfra(91-210) and were unambiguously assigned to residues 82-90 (the resonance of residue 83 is missing, residue 87 is a Pro). On average, they are less intense than the other resonances. Some also have a clear pH dependence and are absent above pH 7. This suggests that the corresponding residues are exposed to the solvent and are flexible, as is typical for unstructured regions.

The spectra of the longer constructs are superposable with that of hfra(81-210) with no evidence of additional peaks, strongly suggesting that further elongation does not contribute to structuring the N-terminus (data not shown). If visible at all, the resonances of the additional residues must either be hidden in the crowded regions of the spectrum, which is typical of random coil conformations, or in intermediate exchange with the solvent, as expected for a flexible and unstructured chain.

### The N-terminal tail is intrinsically unfolded

**Fig. 3:**
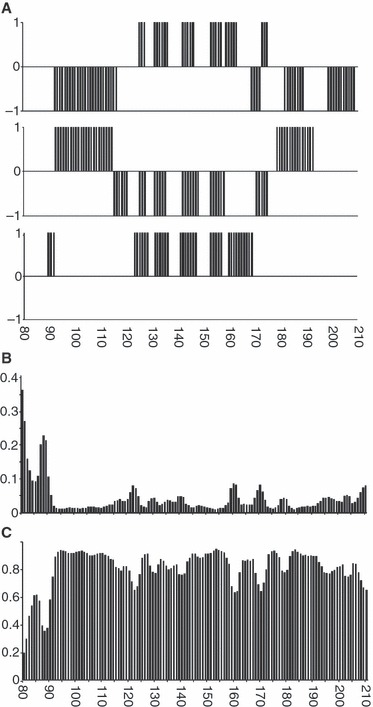
Assessment of the dynamic properties of hfra(81-210) as calculated from the chemical shift values. (A) From top to bottom: chemical shift indices of Hα, Cα and Cβ, calculated as described previously [23]. (B) Random coil index and (C) order parameters [24].

Analysis of the chemical shifts with various predictive tools to estimate the degree of structure of the N-terminal extension provided further evidence that the N-terminal tail is intrinsically unfolded. Analysis of the secondary chemical shifts using chemical shift indices [23] supports the hypothesis of am absence of secondary structure in the region 81-92 (Fig. 3A). As a control, this method correctly predicted the positions of the secondary structure elements in the domain.

Finally, we used random coil index analysis to estimate the flexibility of the N-terminal extension in terms of the root mean square fluctuations of structure ensemble from the experimental chemical shifts (Fig. 3B) and order parameters (Fig. 3C) [24]. This analysis provides a semi-quantitative means to combine the chemical shift values from six nuclei (^13^Cα, ^13^Cβ, ^13^CO, ^15^N, HN and Hα) into a single parameter that correlates with the amplitudes of backbone protein motions. Both plots strongly support high flexibility of the N-terminus.

### Relaxation experiments show that the N-terminus is flexible

**Fig. 4:**
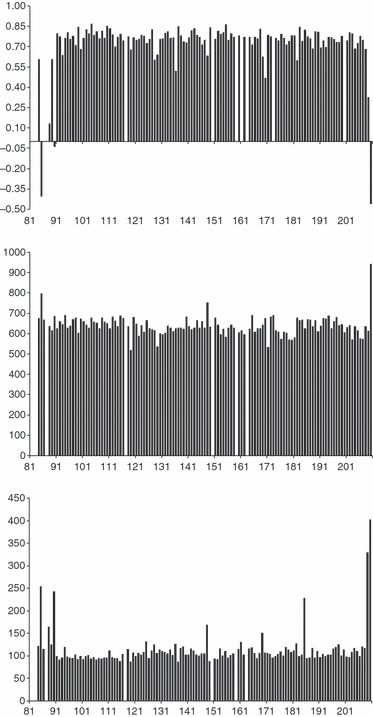
NMR relaxation parameters. From top to bottom, the three panels represent the NOE, T_1_, and T_2_ values, respectively, as measured at 600 MHz and 25 °C.

A more direct analysis of the dynamics was achieved by measuring the NMR relaxation parameters (Fig. 4). The experimental T_1_ values range from 518.1 to 941.0 ms. The maximal value was observed for the C-terminal residue Ala210. If this residue is omitted, the mean T_1_ is 635.8 ms. The T_2_ values range from 86.1 to 402.0 ms, with Ala210 again having the largest value. The mean T_2_ value, excluding this residue, is 111.4 ms. Negative NOE values were observed for the N-terminal residues and for the first Gly in the GHPG motif, which has an unusual chemical shift in the proton dimension [25]. Its negative NOE value can almost certainly be explained by its proximity to the water signal, and therefore by the effect of water suppression.

### The N-terminal tail does not alter the iron-binding properties of hfra

**Fig. 5:**
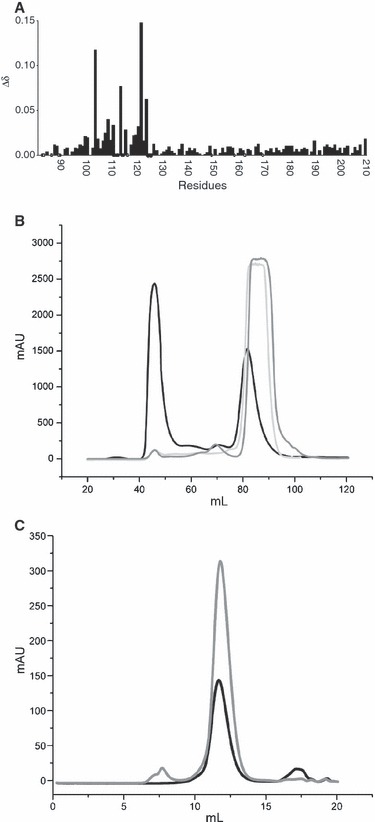
Probing the iron-binding and aggregation properties of hfra(81-210). (A) Histogram of the chemical shift perturbation observed when titrating hfra(81-210) with two molar equivalents of Fe^2+^. Filled circles correspond to residues whose resonances disappear, open circles are used to indicate positions (including prolines) for which the HSQC resonance is missing. (B) Gel filtration elution profile of hfra(60-210) (black line), hfra(81-210) (light grey) and hfra(91-210) (dark grey) using a preparative Superdex 75 16/60 column (GE Healthcare). (C) Gel filtration elution profile of hfra(81-210) in the presence (black line) and absence (grey line) of Fe^2+^ using a pre-packed analytical HiLoad 10/30 Superdex 75 column (GE Healthcare).

To check whether the unstructured tail influences the iron-binding properties of hfra, we titrated hfra(81-210) both by Fe^2+^ and Fe^3+^ using NMR as the detection tool. As previously observed [26], anaerobic Fe^2+^ titration led to disappearance of the amide resonances of residues 112, 113, 115, 125 and 126, and shift and/or broadening of residues 104, 109, 111, 114, 122 and 124 at a 1:2 protein:iron molar ratio (Fig. 5A). When hfra(81-210) was titrated with Fe^3+^, we observed visible precipitation, suggesting that, at neutral pH, precipitation of the ferric hydroxide competes with binding and predominates at these concentrations (data not shown). This is in agreement with the weak binding constant found previously [8]. We obtained similar results with the longer constructs (data not shown).

### The N-terminal tail does not alter the monodispersity of hfra

We then compared the aggregation properties of our constructs. We noticed that, during purification by gel filtration on a Superdex 75 column, hfra(61-210) runs as two distinct species. One corresponds to the monomer, while the second is present in the excluded volume of the column and corresponds to a large hfra aggregate, as supported by SDS–PAGE analysis (Fig. 5B). No such behaviour was observed with hfra(81-210), suggesting that the additional residues could be responsible for previous results showing that hfra can aggregate under specific conditions [27].

To investigate the ability of the extended proteins to aggregate in the presence of Fe^2+^, we studied the chromatographic profile of hfra(81-210) in the presence of a 20-fold excess of Fe(NH_4_)_2_(SO_4_)_2_ (Fig. 5C, dark line). For comparison, the elution profile of the apoprotein is shown (Fig. 5C, grey line). In both cases, we detected the peak corresponding to the monomeric form but no high-molecular-weight aggregates, although they were observed in similar experiments for the bacterial and yeast orthologues CyaY and Yfh1 [28,29]. These results confirm that hfra is not prone to iron-induced aggregation, as also observed for the isolated globular domain [28].

## Discussion

We have addressed here the question of whether the N-terminal tail that is present only in eukaryotic frataxins is structured, taking the human protein as a model. While flexibility of this region was suggested by the absence of a detectable density map for residues 84-89 in the crystal structure of the hfra(84-210) construct [13] and by the absence of stabilizing/destabilizing effects of N-terminal extensions on the thermodynamic stability of the evolutionary conserved domain [11], no direct information was available for what we now know to be the mature hfra [17,18]. The equivalent region of the yeast orthologue appears to adopt a relatively well-defined conformation in both the NMR [14] and X-ray structures [15]. Here we provide complementary evidence showing that the N-terminal tail of hfra is unstructured and flexible, thus indicating that the region is intrinsically unfolded.

The concept of intrinsically unfolded proteins, as opposed to the widespread traditional view that proteins must have a fixed tertiary structure, has recently attracted much interest [30]. This has led to the realization that a large number of soluble proteins are either completely unstructured or contain unstructured regions. The flexibility of these tracts is often necessary for their functions through an induced fit mechanism that occurs only upon molecular interactions.

What could then be the role of the non-conserved N-terminal tail in the function of human frataxin? While some conservation is observed among mammals, the sequence is rather divergent when considering the whole eukaryotic family. This indicates that, if it takes part of any process at all, the extension could contribute to specific species adaptations. The corresponding N-terminal tail of yeast frataxin, which contains a 3_10_ helix, was suggested to stabilize a trimer [31]. This oligomer was thought to be the functional unit for iron transferring to its partners and the building block for frataxin oligomerization [32]. At variance with these results, we have previously shown that the N-terminal tail does not contribute to protein stability [11]. In this work, we provide direct evidence that the tail (in all degrees of elongation studied here) does not alter the structure, the iron-binding properties or the monodispersity of the evolutionarily conserved domain even in the presence of high concentrations of iron.

We propose that the role of the N-terminal tail in mature hfra, which is shorter than previously suggested [17,18,33], is either to take part in and stabilize the interactions with other protein partners (e.g. the Nfs1 desulfurase or ferrochelatase), or, more simply, to act as a spacer that is necessary to allow correct processing of the protein by the MPP protease. It is reasonable to assume that, if the cleavage site were too close to the compact and rather rigid evolutionarily conserved domain, interaction with MPP could be difficult and cleavage less efficient. The latter hypothesis may explain the poor conservation of this region. More studies are required to test this working hypothesis.

## Experimental procedures

### Bioinformatics tools

Eukaryotic frataxin sequences were retrieved by a BLASTp search (http://blast.ncbi.nlm.nih.gov) [19] and aligned using Clustal X version 2.0 (http://www.clustal.org) [20]. Intrinsically unfolded regions were predicted using the DISEMBL (http://dis.embl.de/) [21] and PONDR (http://www.pondr.com/) [22] web servers. Chemical shift analysis was carried out using the CSI (chemical shift indices) (http://www.bionmr.ualberta.ca/bds/software/csi/latest/csi.html) [23] and RCI (random coil index) (http://wishart.biology.ualberta.ca/rci/cgi-bin/rci_cgi_1_e.py) [24] web servers.

### Protein production

The constructs used span the sequence of hfra (residues 61-210 and 81-210 of Genbank accession number U43752). The proteins were produced as His-tagged fusion constructs cloned into a pET9-derived plasmid vector as previously described [11]. In brief, the constructs were expressed in *Escherichia coli* strain BL21 (DE3), transformed with modified pET vector DNA, and purified. The cells were inoculated into Luria broth (LB) medium with kanamycin (30 mg·L^−1^) and induced using isopropyl thio-β-d-thiogalactoside (IPTG). Ni-NTA chromatography was used as the first step of purification, using imidazole for elution. Final purification was achieved by gel filtration chromatography on a Superdex 75 16/60 column (GE Healthcare, Chalfont St Giles, UK). The purity of the recombinant proteins was checked by SDS–PAGE after each step of purification and by mass spectrometry on the final products. Complete anti-protease cocktail (Roche, Basel, Switzerland) was used to prevent proteolytic cleavage of hfra(61-210).

^15^N- and ^15^N,^13^C-labelled samples were produced by growing the bacteria in minimal medium using (^15^NH_4_)_2_SO_4_ and ^13^C_6_-d-glucose as sole sources of nitrogen and carbon, respectively.

### NMR measurements

The experiments were performed at 25 °C on a Varian (Palo Alto, CA, USA) Inova 600 MHz NMR spectrometer equipped with a triple-resonance gradient probe. Protein samples (0.3–1 mm) were prepared in either 10 mm sodium phosphate buffer at pH 7.0 or 8.0 or 50 mm Tris/HCl, 10 mm NaCl at pH 7.2 with 10% D_2_O. Assignment of the region 91-210 was retrieved from the BMRB database (entry number 4342). Assignment of the resonances of residues in the N-terminal extension was obtained by standard methods using HNCA, HNCOCA and ^15^N NOESY-HSQC experiments.

Thirteen data points were collected for T_1_ and T_2_ measurements, using delays of 1.5, 49.6, 97.8, 145.9, 194.1, 250.3, 298.4, 394.8, 499.0, 747.9, 996.7, 1245.5 and 1494.3 ms and 0, 8.7, 17.4, 26.1, 34.7, 43.4, 52.1, 60.8, 69.5, 86.9, 104.2, 121.6 and 147.7 ms, respectively. T_1_ and T_2_ spectra were recorded as 256 × 1024 (440 × 2048) complex data points with 16 scans. Spectral widths were 1800 and 6500 Hz along ^15^N and ^1^H dimensions, respectively. Heteronuclear NOE spectra were recorded as 128 × 1024 complex data points with 48 scans. The spectral widths for ^15^N and ^1^H were as given above. NOE values were determined as the ratio of peak intensity in the absence and the presence of proton saturation.

Ferrous and ferric ammonium sulfate salts (Sigma, St Louis, MO, USA) were used for iron titrations, which were performed under anaerobic conditions for Fe^2+^, using a Belle chamber (Belle Technology, Weymouth, UK) under nitrogen atmosphere, and under aerobic conditions for Fe^3+^.

All spectra were processed by nmrpipe. Intensities were extracted using non-linear spectral lineshape modelling, and fit to single exponentials using routines within NMRPipe [34].

#### Aggregation studies

A pre-packed HiLoad 10/30 Superdex 75 column (GE Healthcare) was equilibrated with Hepes buffer (pH 7.4) and 150 mm NaCl. Samples (1 mL) were loaded using a static loop (1 mL), and were eluted with the same equilibrating buffer. Samples were prepared by incubating 20 μm protein in a 20-fold excess of Fe(NH_4_)_2_(SO_4_)_2_ for 1 h at 30 °C. After centrifugation at 13 000 ***g*** for 5 min, the supernatant was loaded on the column.

## Abbreviation

hfra: human frataxin
